# Alterations in Rat Accumbens Endocannabinoid and GABA Content during Fentanyl Treatment: The Role of Ghrelin

**DOI:** 10.3390/ijms18112486

**Published:** 2017-11-22

**Authors:** Magdalena Sustkova-Fiserova, Chrysostomos Charalambous, Tereza Havlickova, Marek Lapka, Pavel Jerabek, Nina Puskina, Kamila Syslova

**Affiliations:** 1 Department of Pharmacology, Third Faculty of Medicine, Charles University, Ruska 87, 100 34 Prague 10, Czech Republic; chrys.chs@gmail.com (C.C.); terez.hav@gmail.com (T.H.); marek.lapka@centrum.cz (M.L.); pa.jerabek@gmail.com (P.J.); 2 Department of Addictology, First Faculty of Medicine, Charles University, Apolinarska 4, 128 00 Prague 2, Czech Republic; nina.puskina@seznam.cz; 3 Laboratory of Medicinal Diagnostics, Department of Organic Technology ICT, Technicka 5, 166 28 Prague 6, Czech Republic; kamila.syslova@vscht.cz

**Keywords:** fentanyl, ghrelin, endocannabinoids, anandamide, 2-arachidonoylglycerol, GABA, neural reward system, nucleus accumbens shell, ventral tegmental area, microdialysis

## Abstract

The opioid-induced rise of extracellular dopamine, endocannabinoid anandamide and γ-aminobutyric acid (GABA) concentrations triggered by opioids in the nucleus accumbens shell (NACSh) most likely participate in opioid reward. We have previously demonstrated that systemic administration of ghrelin antagonist (JMV2959) significantly decreased morphine-induced dopamine and anandamide (*N*-arachidonoylethanolamine, AEA) increase in the NACSh. Fentanyl is considered as a µ-receptor-selective agonist. The aim of this study was to test whether JMV2959, a growth hormone secretagogue receptor (GHS-R1A) antagonist, can influence the fentanyl-induced effects on anandamide, 2-arachidonoylglycerol (2-AG) and GABA in the NACSh and specify the involvement of GHS-R1A located in the ventral tegmental area (VTA) and nucleus accumbens (NAC). Using in vivo microdialysis in rats, we have found that pre-treatment with JMV2959 reversed dose dependently fentanyl-induced anandamide increases in the NACSh, resulting in a significant AEA decrease and intensified fentanyl-induced decreases in accumbens 2-AG levels, with both JMV2959 effects more expressed when administered into the NACSh in comparison to the VTA. JMV2959 pre-treatment significantly decreased the fentanyl-evoked accumbens GABA efflux and reduced concurrently monitored fentanyl-induced behavioural stimulation. Our current data encourage further investigation to assess if substances affecting GABA or endocannabinoid concentrations and action, such as GHS-R1A antagonists, can be used to prevent opioid-seeking behaviour.

## 1. Introduction

Gut-brain orexigenic peptide ghrelin [1], a natural ligand of the growth hormone secretagogue receptor (GHS-R1A), has been recently shown to play a critical role in food reward [2] as well as reward, motivation and intake of alcohol and reward of several stimulants (for review, see [3,4]). In addition to hypothalamus, the central GHS-R1As are expressed in important reward related areas including striatum, nucleus accumbens (NAC), amygdala, prefrontal cortex, hippocampus, and ventral tegmental area (VTA) [5,6,7,8,9,10,11,12]. Available literature involving ghrelin in opioid abuse and addiction is still limited and inconclusive [13,14,15,16,17]. The self-administration study of Maric et al. [13] showed that ghrelin administered intracerebroventricularly (i.c.v.) could increase heroin intake, however, pre-treatment with a peptide GHS-R1A antagonist (i.c.v.) did not affect heroin self-administration. However, we have shown in our earlier study [16] that premedication with the GHS-R1A antagonist, a triazole non-peptidic derivative JMV2959 [18], significantly and dose-dependently reduced morphine-induced dopamine efflux in the nucleus accumbens shell (NACSh), a brain structure that is crucially important for drug reward mediation [19,20], and attenuated behavioural stimulation, particularly stereotypical behaviours induced by morphine. This was subsequently confirmed in mice [15]. Engel et al. [15] also described that JMV2959 significantly reduced expression of morphine-induced conditioned place preference (CPP) in mice and we have recently documented the same in rats [21]. Conclusively, this suggests a significant involvement of central ghrelin system in changes induced by morphine/opioids in the mesolimbic dopaminergic system, changes which are associated with processing of neural reward.

The opioid/µ-receptor agonists’ rewarding reinforcing properties are traditionally associated with opioid-induced accumbens dopamine efflux [22,23,24,25] caused by supressing γ-aminobutyric acid (GABA) release from VTA interneurons, which tonically inhibit mesolimbic dopamine neurons [26,27,28]. However, intra-accumbens opioid administration also induced dopamine increase [29,30,31] and inhibited accumbens fast amino-acid-mediated synaptic transmission [32,33,34]. Opioids are self-administered into the NACSh as well as VTA [28,35]. Thus, at least part of the opioid rewarding effects may be due to their direct or indirect effect on synaptic transmission in the NAC. Both dopaminergic and non-dopaminergic circuits can contribute to VTA and NAC opioid reward [28,36].

The endogenous cannabinoid system provably significantly participates in reinforcing processes of opioids [37,38,39,40,41,42]. Cannabinoid CB1 receptor (CB1) antagonists such as SR141716A (rimonabant) reduced the opioid rewarding effects in both conditioned place preference models [43,44,45] and intravenous self-administration [24,44,46,47], whereas δ-9-tetrahydrocannabinol (THC), a CB1 agonist, increased the reinforcing effects of the intravenously self-injected heroin [48]. Caille and Parsons [49] documented that intravenous self-administration of heroin was significantly attenuated by CB1 antagonist SR141716A infused into the NACSh, thence CB1 receptors possibly modulate reward of opioids through the ventral striatopallidal projection. There is also evidence of cannabinoid–opioid interaction mediated by activation of presynaptic CB1 and µ-receptors (colocalization or heterodimerization) of the same or linked accumbens neurons, particularly NACSh, similar to the neurons within the VTA [37,50]. However, systemic SR141716A pre-treatment had no significant effect on morphine-induced dopamine increases in the NACSh [46,51]. It follows that CB1 receptors in the NACSh significantly modulate opioid reinforcement through dopamine-independent mechanisms [52]. The contemplated role of the CB2 recently discovered in the VTA in reward is not yet clear [53].

It has also been described [52] that during the heroin self-administration *N*-arachidonoylethanolamine (anandamide, AEA) levels were significantly enhanced and simultaneously 2-arachidonoylglycerol (2-AG) levels significantly decreased in the dialysates from the NACSh. This is in accordance with our previous microdialysis study in rats [17] where we observed a significant AEA increase and significant 2-AG decrease in the NACSh dialysates after acute morphine dose as well as when morphine was administered during prolonged abstinence from repeated morphine. In addition, Vigano et al. [54] measurements of accumbens 2-AG and AEA contents post mortem after sub-chronic morphine administration in rats showed AEA increase and 2-AG decrease. Thus, the endocannabinoids input in the opioid motivational properties was supported, possibly particularly AEA increase induced by opioids in the NACSh contributes to opioid reward [52].

The most-characterized endocannabinoids are anandamide (AEA) [55] and 2-AG [56,57]. AEA and 2-AG differ in many aspects, they are formed in various brain structures under different conditions and are uniquely affected by different stimuli, including pharmacological interference [58,59,60,61]. Endocannabinoids are formed “on demand” and they act as retrograde messengers in the central nervous system (CNS) through activation of presynaptic CB1 receptors on both excitatory and inhibitory synapses [59,62,63,64]. Endocannabinoids, which are released after depolarization in the NAC and from VTA dopaminergic neurons, possibly modulate glutamatergic and GABAergic afferents as retrograde messengers on different neuronal receptors [65]. As explained earlier, opioids trigger the release of AEA in the NACSh, which is possibly participating in opioid reward in a dopamine-nondependent manner [52], however, compounds that increased brain concentrations of AEA and prolonged AEA’s effects did not influence heroin self-administration in rats, thus the importance of this release is still rather unclear [48]. Thus far, we have even more limited knowledge about the role of 2-AG in opioid reward processes in the NACSh.

In our previous study, we have documented a significant interaction between ghrelin and endocannabinoids in the morphine-induced changes in the NACSh [17]. Premedication with JMV2959 before morphine, which was administered in acute doses or in a challenge dose during prolonged abstinence from chronic morphine, dose-dependently reversed the morphine-induced AEA increase in the NACSh leading to a significant AEA drop. JMV2959 intensified significantly the acute morphine-induced decrease in 2-AG concentrations and reduced challenge morphine induced 2-AG decrease in the NACSh. Besides our results, we have found several studies substantiating relevant interactions between ghrelin and cannabinoid systems in the food intake regulation by the brain-gut axis [66,67,68,69,70,71,72]. Fentanyl, a 4-anilidopiperidin derivate synthetized in 1959 [73], is generally considered as a µ-receptor-selective agonist about 100-fold more potent than morphine [74], using active transportation through the blood–brain barrier [75,76]. Fentanyl and new opioid synthetic derivatives have been recently increasingly abused in the USA, Canada and Europe [77]. The goal of the present study was to establish whether the GHS-R1A antagonist, the substance JMV2959 could influence the fentanyl-induced effects on anandamide and 2-AG in the NACSh and specify the involvement of GHS-R1A located in the VTA and NAC.

Opioid/morphine administered systemically or into the NAC can also stimulate accumbens GABA efflux [78,79] and there is evidence about its contribution to the opioid reinforcing properties [29,80]. It has been suggested that the simultaneous activation of µ and GABA-A receptors which are co-expressed on GABAergic interneurons significantly supress GABA efflux onto dopamine nerve endings, disinhibits dopamine neurons and enhances dopamine efflux [29], thus elevated GABA concentrations potentiate opioid-induced dopamine release in the NAC. However, the VTA/NAC dopamine neurons can co-release other modulators such as endocannabinoids, GABA, glutamate, and variety of neuropeptides, which may contribute to the behavioural consequences of inhibiting or stimulating “dopamine” neurons. Various dopamine non-dependent mechanisms, where GABA is supposed to play an important role, also participate in opioid reinforcing processes [28,81]. CB1 and µ receptors are located on GABA neurons in several brain areas including VTA and NAC [42,82]. Recently, it has been suggested how important is the role of ghrelin system in regulation of GABAergic transmission in the central nucleus of amygdala (CeA) together with a complex interaction of ghrelin and ethanol at CeA GABAergic synapses [83]. GHS-R1A activation also attenuated hypothalamic GABA release [84]. Therefore, another goal of our present study was to test the influence of ghrelin antagonism on the fentanyl-induced accumbens GABA efflux and specify the involvement of GHS-R1A located in the VTA and NAC. To get a more complex picture, we have monitored also behavioural changes in rats during the microdialysis experiment, when ghrelin antagonist was administered intraperitoneally before fentanyl.

## 2. Results

### 2.1. The Effects of Growth Hormone Secretagogue Receptor (GHS-R1A) Receptor Antagonist on Fentanyl-Induced Accumbens Anandamide (AEA) Extracellular Concentration Increase

#### 2.1.1. Pre-Treatment with Intraperitoneal JMV2959

**Figure 1:**
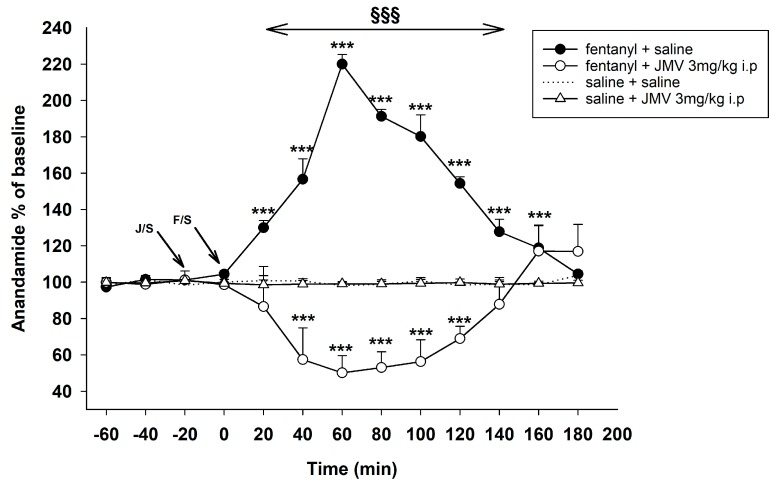
Effects of ghrelin receptor antagonist JMV2959 3 mg/kg administered intraperitoneally (i.p.) on the fentanyl-induced accumbens anandamide (AEA) levels. JMV2959 was administered following three 20 min baselines and 20 min before fentanyl/saline (intervals: baseline = −60 to −20 min; JMV2959 pre-treatment = 0 min; fentanyl = 20–180 min) (means ± SEM). The effects are illustrated as follows: saline + fentanyl (filled circle), 3 mg/kg JMV2959 + fentanyl (open circle), 3 mg/kg JMV2959 + saline (open triangle), saline + saline (dotting). Differences to saline + saline group are expressed as *** *p* < 0.001. Differences between fentanyl + saline and 3 mg/kg JMV2959 + fentanyl effects are expressed as ^§§§^ *p* < 0.001. The horizontal arrow shows intervals with appropriate significant changes (^§§§^); the oblique arrows show the time of administration of J/S = JMV2959/saline and F/S = fentanyl/saline.

The influence of intraperitoneally administered ghrelin antagonist on fentanyl-induced increase of extracellular AEA in the NACSh is illustrated in Figure 1. Baseline levels of AEA did not significantly differ between animals in all presented experiments. As expected, acute systemic fentanyl (30 µg/kg subcutaneous—s.c.) administration evoked a statistically significant efflux of AEA in the NACSh. The two-way ANOVA for repeated measures (RM) followed by Bonferroni’s multiple comparisons procedure has shown a significant group effect: saline + fentanyl 30 µg/kg vs. saline + saline group (F1,10 = 813.8, *p* < 0.001) and time effect (F10,100 = 81.9, *p* < 0.001); time course of AEA changes in the NACSh after saline/fentanyl injection differed significantly between the two groups of rats (time × group interaction, F10,100 = 86.8, *p* < 0.001). The fentanyl-induced AEA increase reached the maximum effect 220% of baseline mean level 60 min after fentanyl administration (*p* < 0.001).

Pre-treatment with the GHS-R1A antagonist JMV2959 administered intraperitoneally (i.p.) turned the fentanyl-induced accumbens AEA increase and induced a significant decrease with the maximum drop 50% of baseline mean level. Thus, the JMV2959 pre-treatment effect was highly statistically significant: JMV2959 3 min/kg + fentanyl 30 µg/kg vs. saline + fentanyl 30 µg/kg: effect of group F1,10 = 217.3, *p* < 0.001; effect of time F10,100 = 7.9, *p* < 0.001; time × group interaction F10,100 = 78.0, *p* < 0.001. Observed changes within the JMV2959 pre-treatment group in comparison to baseline were also significant (*p* < 0.001) (JMV2959 3 mg/kg + saline vs. saline + saline: effect of group F1,10 = 18.7, *p* < 0.01; effect of time F10,100 = 14.9, *p* < 0.001; time × group interaction F10,100 = 13.7, *p* < 0.001). The JMV2959 pre-treatment induced decrease/reversal of accumbens AEA was observed within about 20–150 min after fentanyl administration, then the AEA levels crossed the baseline levels and reached a significant AEA increase with maximum 117% of baseline.

A single dose of JMV2959 3 mg/kg i.p. had no effect on accumbens AEA and neither for saline i.p.

#### 2.1.2. Pre-Treatment with JMV2959 Administered into the Ventral Tegmental Area (VTA)

**Figure 2:**
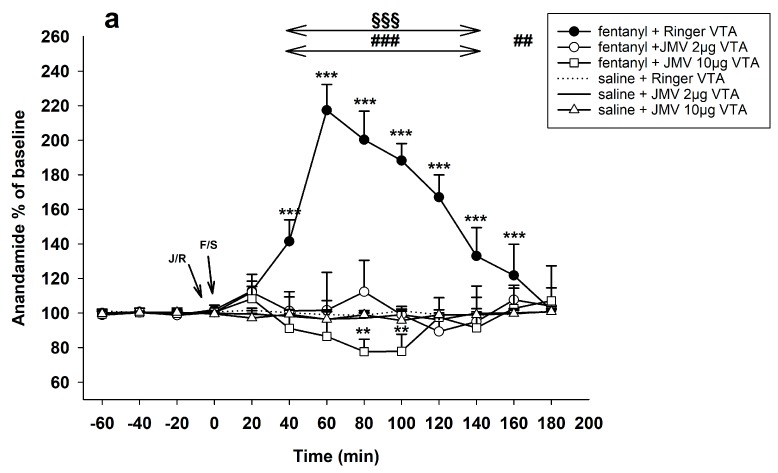

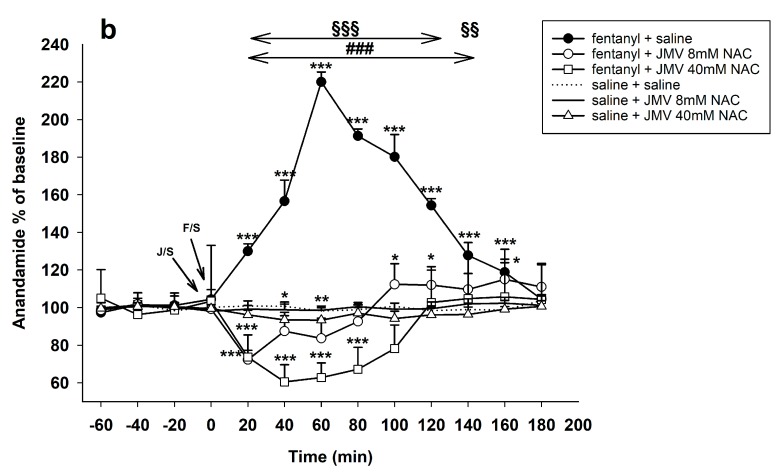
Effects of growth hormone secretagogue receptor (GHS-R1A) antagonist JMV2959 administered following four 20 min baselines into the ventral tegmental area (VTA) or into the nucleus accumbens (NAC) always 5 min before fentanyl/saline on the accumbens AEA levels (means ± SEM). The effects are illustrated as follows: (**a**) Ringer’s solution/VTA + fentanyl (filled circle), 2 µg JMV2959/VTA + fentanyl (open circle), 10 µg JMV2959/VTA + fentanyl (open square), 2 µg JMV2959/VTA + saline (continuous line), 10 µg JMV2959/VTA + saline (open triangle), and Ringer’s solution + saline (dotting); and (**b**) saline + fentanyl (filled circle), JMV2959 lower dose 8 mM/15 min/NAC + fentanyl (open circle), JMV2959 higher dose 40 mM/15 min/NAC + fentanyl (open square), JMV2959 lower dose 8 mM/15 min/NAC + saline (continuous line), JMV2959 higher dose 40 mM/15 min/NAC + saline (open triangle), and Ringer’s solution + saline (dotting). Differences between treatments and the control group (Ringer’s/VTA + saline or saline + saline) are expressed as *** *p* < 0.001, ** *p* < 0.01, * *p* < 0.05. Differences between fentanyl and fentanyl in combinations with the higher JMV2959 dose (10 µg/VTA or 40 mM/15 min/NAC, respectively) are expressed as ^###^ *p* < 0.001, ^##^ *p* < 0.01. Differences between fentanyl and fentanyl in combinations with the lower JMV2959 dose (2 µg/VTA or 8 mM/15 min/NAC, respectively) are expressed as ^§§§^ *p* < 0.001, ^§§^ *p* < 0.01. The horizontal arrows show intervals with appropriate significant changes (^§§§^ or ^###^); the oblique arrows show the time of administration of J/S = JMV2959/saline or J/R = JMV2959/Ringer´s solution and F/S = fentanyl/saline.

Figure 2a illustrates the observed influence of ghrelin antagonist, administered into the VTA, on changes in accumbens AEA induced by 30 µg/kg s.c. fentanyl. The 30 µg/kg dose of fentanyl together with intra-VTA Ringer’s solution induced practically the same AEA increase as the above described fentanyl with systemic saline: Ringer’s solution/VTA + fentanyl 30 µg/kg vs. Ringer’s solution/VTA + saline: effect of group F1,10 = 243.0, *p* < 0.001; effect of time F9,90 = 55.1, *p* < 0.001; time × group interaction F9,90 = 57.8, *p* < 0.001; with maximum effect 217% of baseline level.

JMV2959 pre-treatment into the VTA 5 min before fentanyl significantly and dose-dependently reduced the opioid-induced AEA increase. The lower dose (2 µg) pre-treatment caused a drop of AEA accumbens levels to the baseline concentration and the higher dose (10 µg) even induced significant AEA decrease with the maximum drop 78% of baseline level (*p* < 0.05). For the lower JMV2959 dose: JMV2959 2 µg/VTA + fentanyl 30 µg/kg vs. Ringer’s solution/VTA + fentanyl 30 µg/kg: effect of group F1,10 = 168.7, *p* < 0.001; effect of time F9,90 = 40.5, *p* < 0.01; time × group interaction F9,90 = 42.9, *p* < 0.001. The effects of JMV2959 2 µg/VTA with fentanyl 30 µg/kg on the accumbens AEA did not significantly differ from Ringer’s solution/VTA with saline. For the higher JMV2959 dose: JMV2959 10 µg/VTA + fentanyl 30 µg/kg vs. Ringer’s solution/VTA + fentanyl 30 µg/kg: effect of group F1,10 = 295.2, *p* < 0.001; effect of time F9,90 = 22.9, *p* < 0.001; time × group interaction F9,90 = 54.5, *p* < 0.001. JMV2959 10 µg/VTA + fentanyl 30 µg/kg vs. Ringer’s solution/VTA + saline: effect of group F1,10 = 5.1, *p* < 0.05; effect of time F9,90 = 2.6, *p* < 0.05; time × group interaction F9,90 = 2.2, *p* < 0.05.

A single dose of JMV2959 2 µg as well as 10 µg/VTA, similarly to Ringer’s solution/VTA had no significant effect on accumbens AEA.

#### 2.1.3. Pre-Treatment with JMV2959 Administered into the Nucleus Accumbens (NAC)

Figure 2b illustrates the observed influence of ghrelin antagonist administered into the NACSh on accumbens fentanyl-induced AEA changes. The 30 µg/kg fentanyl effects on accumbens AEA were practically the same with both pre-treatment with saline i.p. and Ringers’s solution into the VTA. Thus, for ethical reasons, we did not create a new group with fentanyl 30 µg/kg without any pre-treatment, but we have used the group with systemic saline pre-treatment instead (see Section 2.1.1) for testing the JMV2959 effects when administered into the NACSh; for statistical evaluation we have used mean of three baselines and results after fentanyl administration (saline + fentanyl 30 µg/kg vs. saline + saline: effect of group F1,10 = 823.5, *p* < 0.001; effect of time F9,90 = 76.0, *p* < 0.001; time × group interaction F9,90 = 81.1, *p* < 0.001; maximum increase 220% of baseline level).

We have used the dialysis probe for administration of JMV2959 into the NAC. After baseline samples were collected, perfusion with Ringer’s solution (2 µL/min) was switched to perfusion with 8 mM or 40 mM JMV2959, respectively, for 15 min, starting 5 min before fentanyl administration; thereafter the inlet tube was switched back to Ringer’s solution. Pre-treatment with both JMV2959 doses into the NACSh dose dependently reversed the fentanyl-induced accumbens AEA increase to a significant decrease with maximum drop 84% of baseline level 60 min after fentanyl (lower JMV2959 dose) and 61% of baseline level 40 min (higher JMV2959 dose) after fentanyl administration, respectively. For the lower JMV2959 dose: JMV2959 8 mM/15 min/NAC + fentanyl 30 µg/kg vs. saline + fentanyl 30 µg/kg: effect of group F1,10 = 120.2, *p* < 0.001; effect of time F9,90 = 46.5, *p* < 0.01; time × group interaction F9,90 = 60.4, *p* < 0.001. For the higher JMV2959 dose: JMV2959 40 mM/15 min/NAC + fentanyl 30 µg/kg vs. saline + fentanyl 30 µg/kg: effect of group F1,10 = 158.8, *p* < 0.001; effect of time F9,90 = 27.0, *p* < 0.01; time × group interaction F9,90 = 70.0, *p* < 0.001. The JMV2959/NAC pre-treatment induced decrease/reversal of accumbens AEA after fentanyl administration were observed only during first intervals, the AEA levels returned to baseline levels at about 90 min (lower JMV2959 dose) and 120 min (higher JMV2959 dose) after fentanyl administration. The lower JMV2959 dose even reached significant accumbens AEA increase within the last one and half hour with maximum 120% of baseline level. (JMV2959 8 mM/15 min/NAC + fentanyl 30 µg/kg vs. saline + saline: effect of group not significant—n.s.; effect of time F9,90 = 20.8, *p* < 0.001; time × group interaction F9,90 = 21.5, *p* < 0.001) (JMV2959 40 mM/15 min/NAC + fentanyl 30 µg/kg vs. saline + saline: effect of group F1,10 = 7.8, *p* < 0.05; effect of time F9,90 = 16.0, *p* < 0.001; time × group interaction F9,90 = 16.2, *p* < 0.001).

Administration of single lower 8 mM JMV2959 dose into the NAC and saline i.p. did not significantly influence the accumbens AEA. Administration of single higher 40 mM JMV2959 dose induced slight but significant AEA decrease with maximum 94% of baseline (JMV2959 40 mM/15 min/NAC + saline vs. saline + saline: effect of group F1,10 = 13.5, *p* < 0.01; effect of time F9,90 = 2.4, *p* < 0.05; time × group interaction F9,90 = 2.2, *p* < 0.05.

### 2.2. The Effects of GHS-R1A Receptor Antagonist on Fentanyl-Induced Accumbens 2-Arachidonoylglycerol (2-AG) Extracellular Concentration Decrease

#### 2.2.1. Pre-Treatment with Intraperitoneal JMV2959 Administration

**Figure 3:**
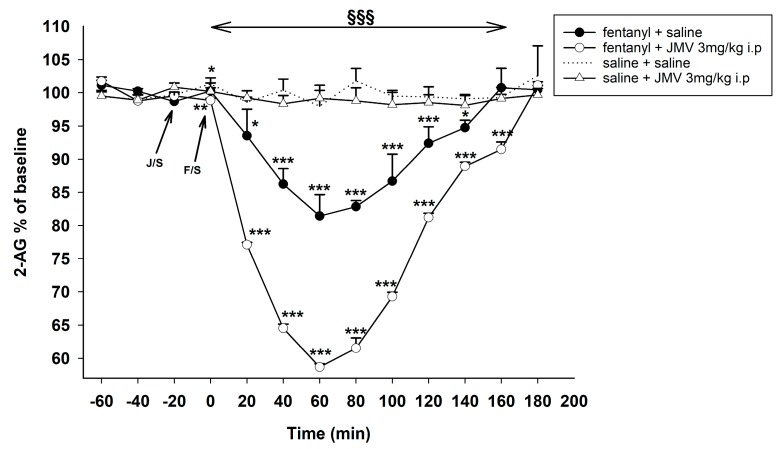
Effects of GHS-R1A antagonist JMV2959 3 mg/kg i.p. on the fentanyl-induced accumbens 2-AG concentration. JMV2959 was administered following three 20 min baselines and 20 min before fentanyl/saline (means ± SEM). The effects are illustrated as follows: saline + fentanyl (filled circle), 3 mg/kg JMV2959 + fentanyl (open circle), 3 mg/kg JMV2959 + saline (open triangle), saline + saline (dotting). Differences to saline + saline group are expressed as *** *p* < 0.001, ** *p* < 0.01, * *p* < 0.05. Differences between fentanyl + saline and 3 mg/kg JMV2959 + fentanyl effects are expressed as ^§§§^ *p* < 0.001. The horizontal arrow shows intervals with appropriate significant changes (^§§§^); the oblique arrows show the time of administration of J/S = JMV2959/saline and F/S = fentanyl/saline.

The influence of intraperitoneally administered ghrelin antagonist on fentanyl-induced 2-AG decrease in the NACSh is illustrated in Figure 3. Baseline extracellular concentrations of 2-AG did not significantly differ between animals in all presented experiments. Fentanyl administration induced a statistically significant decrease of accumbens shell 2-AG with maximum drop 81% of baseline level 1 h after fentanyl administration: saline + fentanyl 30 µg/kg vs. saline + saline: effect of group F1,10 = 197.6, *p* < 0.001; effect of time F10,100 = 18.4, *p* < 0.001; time × group interaction F10,100 = 16.0, *p* < 0.001.

Pre-treatment with JMV2959 intensified the fentanyl-induced accumbens 2-AG decrease. The 3 mg/kg i.p. JMV2959 pre-treatment significantly deepened the fentanyl-induced 2-AG drop into maximum 59% of baseline level: JMV2959 3 mg/kg + fentanyl 30 µg/kg vs. saline + fentanyl 30 µg/kg: effect of group F1,10 = 566.1, *p* < 0.001; effect of time F10,100 = 246.0, *p* < 0.001; time × group interaction F10,100 = 33.1, *p* < 0.001. (JMV2959 3 mg/kg + fentanyl 30 µg/kg vs. saline + saline: effect of group F1,10 = 391.4, *p* < 0.001; effect of time F10,100 = 321.5, *p* < 0.001; time × group interaction F10,100 = 290.7, *p* < 0.001).

JMV2959 3 mg/kg i.p. administered in a single dose did not significantly influence the accumbens 2-AG, also saline i.p. had no effect on 2-AG in the NACSh.

#### 2.2.2. Pre-Treatment with JMV2959 Administered into the VTA

**Figure 4:**
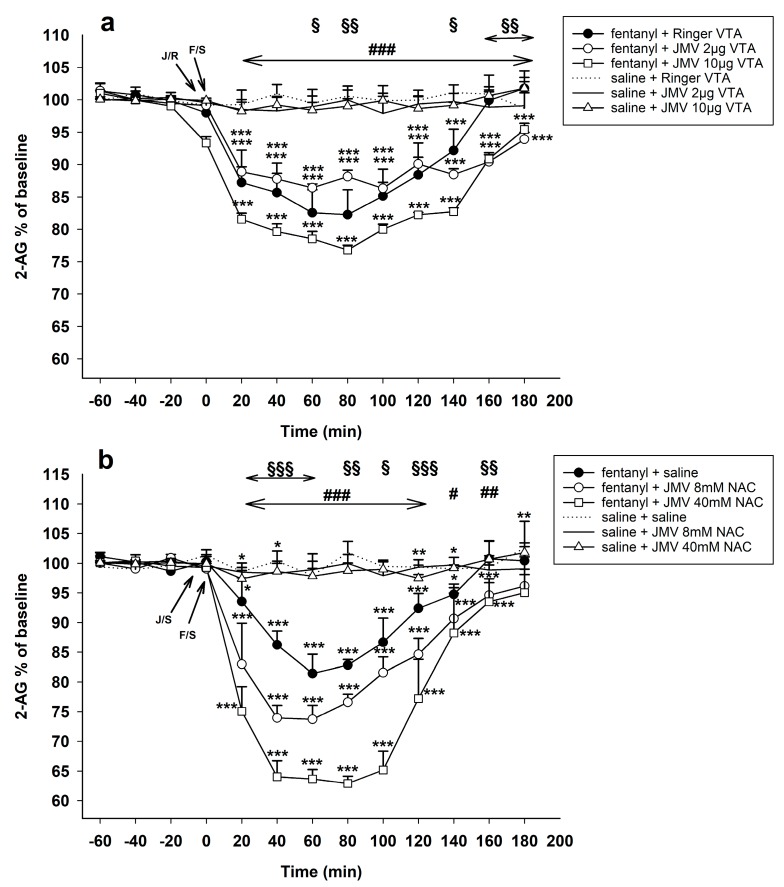
Effects of ghrelin receptor antagonist JMV2959 administered following four 20 min baselines into the VTA or into the NAC 5 min before fentanyl/saline on the accumbens 2-AG levels (means ± SEM). The effects are illustrated as follows: (**a**) Ringer’s solution/VTA + fentanyl (filled circle), 2 µg JMV2959/VTA + fentanyl (open circle), 10 µg JMV2959/VTA + fentanyl (open square), 2 µg JMV2959/VTA + saline (continuous line), 10 µg JMV2959/VTA + saline (open triangle), and Ringer’s solution + saline (dotting); and (**b**) saline + fentanyl (filled circle), JMV2959 lower dose 8 mM/15 min/NAC + fentanyl (open circle), JMV2959 higher dose 40 mM/15 min/NAC + fentanyl (open square), JMV2959 lower dose 8 mM/15 min/NAC + saline (continuous line), JMV2959 higher dose 40 mM/15 min/NAC + saline (open triangle), and Ringer’s solution + saline (dotting). Differences between treatments and the control group (Ringer’s/VTA + saline or saline + saline) are expressed as *** *p* < 0.001, ** *p* < 0.01, * *p* < 0.05. Differences between fentanyl and fentanyl in combinations with the higher JMV2959 dose (10 µg/VTA or 40 mM/15 min /NAC, respectively) are expressed as ^###^ *p* < 0.001, ^##^ *p* < 0.01, ^#^ *p* < 0.05. Differences between fentanyl and fentanyl in combinations with the lower JMV2959 dose (2 µg/VTA or 8 mM/15 min, respectively) are expressed as ^§§§^ *p* < 0.001, ^§§^ *p* < 0.01, ^§^ *p* < 0.05. The horizontal arrows show intervals with appropriate significant changes (^§§§^ or ^§§^ or ^###^); the oblique arrows show the time of administration of J/S = JMV2959/saline or J/R = JMV2959/Ringer´s solution and F/S = fentanyl/saline.

Figure 4a illustrates the observed influence of ghrelin antagonist administered into the VTA on the fentanyl–induced accumbens 2-AG decrease. The 30 µg/kg dose of fentanyl together with intra-VTA Ringer’s solution induced practically the same 2-AG decrease as the above described fentanyl with systemic saline, with maximum of 82% of baseline level: Ringer’s solution/VTA + fentanyl 30 µg/kg vs. Ringer’s solution/VTA + saline: effect of group F1,10 = 80.0, *p* < 0.001; effect of time F9,90 = 26.7, *p* < 0.001; time × group interaction F9,90 = 28.0, *p* < 0.001.

Pre-treatment with JMV2959 influenced the fentanyl-induced accumbens 2-AG decrease differently depending on the given dose. The 2 µg JMV2959/VTA dose slightly but significantly attenuated and simultaneously also prolonged the fentanyl-induced 2-AG decrease, with maximum 87% of baseline. On the contrary, the 10 µg JMV2959/VTA dose significantly deepened the accumbens fentanyl-induced 2-AG decrease to maximal drop 77% of baseline. For the lower dose: JMV2959 2 µg/VTA + fentanyl 30 µg/kg vs. Ringer’s solution/VTA + fentanyl 30 µg/kg: effect of group n.s.; effect of time F9,90 = 68.3, *p* < 0.001; time × group interaction F9,90 = 13.9, *p* < 0.001. For the higher dose: JMV2959 10 µg/VTA + fentanyl 30 µg/kg vs. Ringer’s solution/VTA + fentanyl 30 µg/kg: effect of group F1,10 = 39.8; effect of time F9,90 = 169.5, *p* < 0.001; time × group interaction F9,90 = 5.1, *p* < 0.001. In comparison to the control group the lower 2 µg/kg JMV2959 dose with fentanyl significantly decreased the accumbens 2-AG: JMV2959 2 µg/VTA + fentanyl 30 µg/kg vs. Ringer’s solution/VTA + saline: effect of group F1,10 = 605.3, *p* < 0.001; effect of time F9,90 = 23.9, *p* < 0.001; time × group interaction F9,90 = 30.0, *p* < 0.001. The higher 10 µg JMV2959 dose with fentanyl, also induced significant 2-AG decrease in the NACSh: JMV2959 10 µg/VTA + fentanyl 30 µg/kg vs. Ringer’s solution/VTA + saline: effect of group F1,10 = 985.9, *p* < 0.001; effect of time F9,90 = 129.1, *p* < 0.001; time × group interaction F9,90 = 142.7, *p* < 0.001.

A single dose of JMV2959 2 µg as well as 10 µg/VTA did not significantly influence the accumbens 2-AG levels, the same as Ringer’s solution/VTA with saline s.c.

#### 2.2.3. Pre-Treatment with JMV2959 Administered into the NAC

Figure 4b illustrates the observed influence of ghrelin antagonist administered into the NACSh on the accumbens fentanyl-induced 2-AG decrease. In addition, the 30 µg/kg fentanyl effects on accumbens 2-AG were practically the same with both, pre-treatment with saline i.p. as well as Ringers’s solution into the VTA. Thus, again, we have used the group with systemic saline pre-treatment of fentanyl (see Section 2.2.1) for testing the JMV2959 effects when administered into the NAC (for statistical evaluation we have used mean of three baselines and fentanyl effects) (saline + fentanyl 30 µg/kg vs. saline + saline: effect of group F1,10 = 196.6, *p* < 0.001; effect of time F9,90 = 17.3, *p* < 0.001; time × group interaction F9,90 = 15.9, *p* < 0.001; maximum decrease 81% of baseline level).

Pre-treatment with both JMV2959 doses into the NAC significantly and dose dependently deepened the fentanyl-induced accumbens 2-AG extracellular concentrations with maximum drop to 74% (lower JMV2959 dose) and 63% of baseline (higher JMV2959 dose), respectively. For lower JMV2959 dose: JMV2959 lower dose 8 mM/15 min/NAC + fentanyl 30 µg/kg vs. saline + fentanyl 30 µg/kg: effect of group F1,10 = 160.9, *p* < 0.001; effect of time F9,90 = 52.6, *p* < 0.001; time × group interaction F9,90 = 2.3, *p* < 0.05. For higher JMV2959 dose: JMV2959 higher dose 40 mM/15 min/NAC + fentanyl 30 µg/kg vs. saline + fentanyl 30 µg/kg: effect of group F1,10 = 79.9, *p* < 0.001; effect of time F9,90 = 80.6, *p* < 0.001; time × group interaction F9,90 = 10.7, *p* < 0.001. Both JMV2959 pre-treatments with fentanyl induced significant 2-AG decrease in comparison to saline + saline: for lower dose: JMV2959 lower dose 8 mM/15 min/NAC + fentanyl 30 µg/kg vs. saline + saline: effect of group F1,10 = 656.3, *p* < 0.001; effect of time F9,90 = 22.2, *p* < 0.001; time × group interaction F9,90 = 23.1, *p* < 0.001; for higher dose: JMV2959 higher dose 40 mM/15 min/NAC + fentanyl 30 µg/kg vs. saline + saline: effect of group F1,10 = 309.4, *p* < 0.001; effect of time F9,90 = 67.7, *p* < 0.001; time × group interaction F9,90 = 68.9, *p* < 0.001.

Single lower JMV2959 dose administered into the NAC and saline i.p. did not significantly influence the accumbens 2-AG. Administration of single higher JMV2959 dose 40 mM/15 min/NAC induced slight but significant 2-AG decrease with maximum 97% of baseline (JMV2959 40 mM/15 min/NAC + saline vs. saline + saline: effect of group F1,10 = 6.6, *p* < 0.05; effect of time F9,90 = 3.1, *p* < 0.01; time × group interaction F9,90 = 3.3, *p* < 0.05.

### 2.3. The Effects of GHS-R1A Receptor Antagonist on Fentanyl-Induced Accumbens γ-Aminobutyric (GABA) Extracellular Concentration Increase

#### 2.3.1. Pre-Treatment with Intraperitoneal JMV2959 Administration

**Figure 5:**
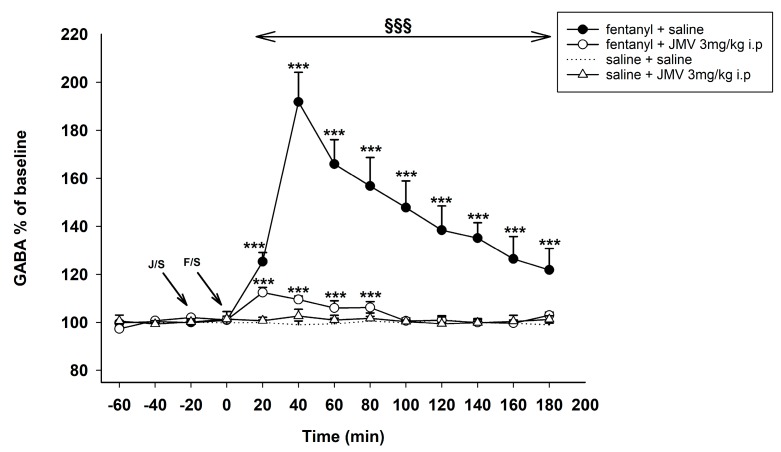
Effects of GHS-R1A antagonist JMV2959 3 mg/kg i.p. on the fentanyl-induced accumbens γ-aminobutyric (GABA) concentration. JMV2959 was administered following three 20 min baselines and 20 min before fentanyl/saline (means ± SEM). The effects are illustrated as follows: saline + fentanyl (filled circle), 3 mg/kg JMV2959 + fentanyl (open circle), 3 mg/kg JMV2959 + saline (open triangle), saline + saline (dotting). Differences to saline + saline group are expressed as *** *p* < 0.001. Differences between fentanyl + saline and 3 mg/kg JMV2959 + fentanyl effects are expressed as ^§§§^ *p* < 0.001. The horizontal arrow shows intervals with appropriate significant changes (^§§§^); the oblique arrows show the time of administration of J/S= JMV2959/saline and F/S = fentanyl/saline.

The influence of intraperitoneally administered ghrelin antagonist on fentanyl-induced increase of accumbens shell extracellular GABA is illustrated in Figure 5. GABA baseline levels did not significantly differ between rats in all presented experiments. As expected, acute systemic fentanyl (30 µg/kg s.c.) administration induced a statistically significant increase of GABA in the NACSh (saline + fentanyl 30 µg/kg vs. saline + saline: effect of group F1,10 = 105.0, *p* < 0.001; effect of time F10,100 = 57.8, *p* < 0.001; time × group interaction F10,100 = 56.4, *p* < 0.001; maximum increase 192% of baseline level).

The GHS-R1A antagonist, JMV2959 i.p. administration 20 min before fentanyl prevented the fentanyl-induced accumbens GABA increase maintaining its concentration almost on the baseline level: JMV2959 3 min/kg + fentanyl 30 µg/kg vs. saline + fentanyl 30 µg/kg: effect of group F1,10 = 158.1, *p* < 0.001; effect of time F10,100 = 65.2, *p* < 0.001; time × group interaction F10,100 = 55.9, *p* < 0.001. Accumbens GABA levels within the JMV2959 + fentanyl group were significantly above the saline levels during the first four intervals after fentanyl (JMV2959 3 mg/kg + saline vs. saline + saline: effect of group F1,10 = 50.1, *p* < 0.05; effect of time F10,100 = 4.6, *p* < 0.001; time × group interaction F10,100 = 4.9, *p* < 0.001; maximum increase 112% of baseline).

A single dose of JMV2959 3 mg/kg i.p. had no effect on accumbens GABA and the same was true for saline.

#### 2.3.2. Pre-Treatment with JMV2959 Administration into the VTA

**Figure 6:**
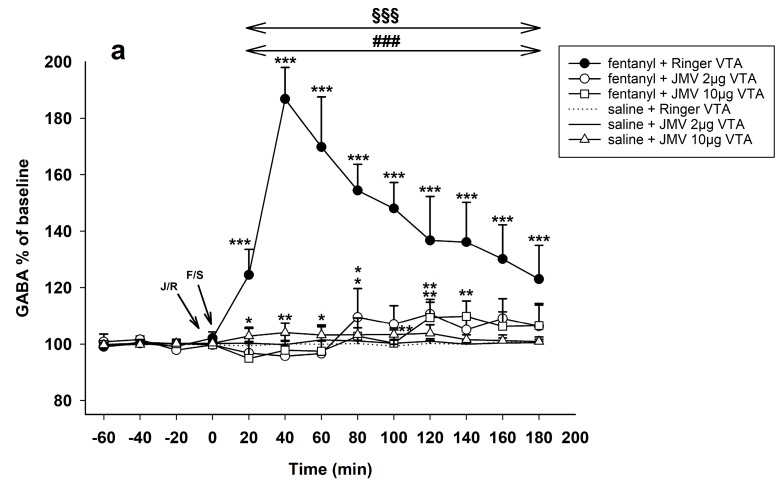

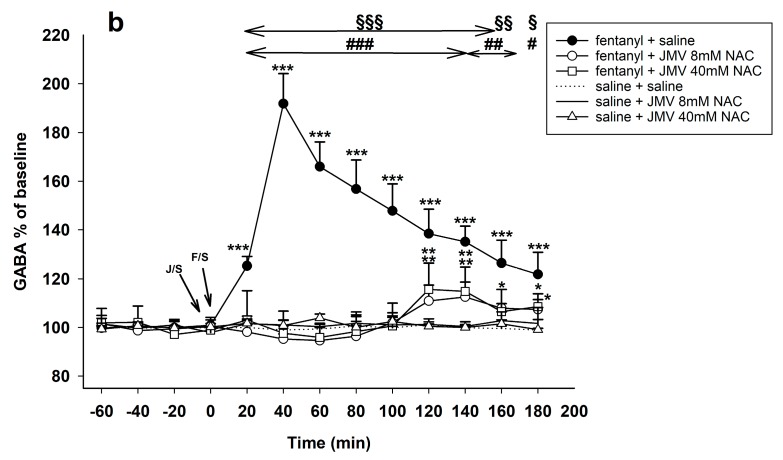
Effects of ghrelin receptor antagonist JMV2959 administered following four 20 min baselines into the VTA and into the NAC 5 min before fentanyl/saline on the accumbens GABA levels (means ± SEM). The effects are illustrated as follows: (**a**) Ringer’s solution/VTA + fentanyl (filled circle), 2 µg JMV2959/VTA + fentanyl (open circle), 10 µg JMV2959/VTA + fentanyl (open square), 2 µg JMV2959/VTA + saline (continuous line), 10 µg JMV2959/VTA + saline (open triangle), and Ringer’s solution + saline (dotting); and (**b**) saline + fentanyl (filled circle), JMV2959 lower dose 8 mM/15 min/NAC + fentanyl (open circle), JMV2959 higher dose 40 mM/15 min/NAC + fentanyl (open square), JMV2959 lower dose 8 mM/15 min/NAC + saline (continuous line), JMV2959 higher dose 40 mM/15 min/NAC + saline (open triangle), and Ringer’s solution + saline (dotting). Differences between treatments and the control group (Ringer’s/VTA + saline or saline/saline) are expressed as *** *p* < 0.001, ** *p* < 0.01, * *p* < 0.05. Differences between fentanyl and fentanyl in combinations with the higher JMV2959 dose (10 µg/VTA or 40 mM/15 min/NAC, respectively) are expressed as ^###^ *p* < 0.001, ^##^ *p* < 0.01, ^#^ *p* < 0.05. Differences between fentanyl and fentanyl in combinations with the lower JMV2959 dose (2 µg/VTA or 8 mM/15 min/NAC, respectively) are expressed as ^§§§^ *p* < 0.001, ^§§^ *p* < 0.01, ^§^ *p* < 0.05. The horizontal arrows show intervals with appropriate significant changes (^§§§^ or ^##^ or ^###^); the oblique arrows show the time of administration of J/S = JMV2959/saline or J/R = JMV2959/Ringer´s solution and F/S = fentanyl/saline.

Figure 6a illustrates the observed influence of ghrelin antagonist administered into the VTA on changes in accumbens GABA induced by 30 µg/kg s.c. fentanyl. The 30 µg/kg dose of fentanyl together with intra-VTA Ringer’s solution again induced practical the same GABA increase as the above described fentanyl with systemic saline: Ringer’s solution/VTA + fentanyl 30 µg/kg vs. Ringer’s solution/VTA + saline: effect of group F1,10 = 65.3, *p* < 0.001; effect of time F9,90 = 11.0, *p* < 0.001; time × group interaction F9,90 = 11.0, *p* < 0.001; with maximum effect 188% of baseline level.

JMV2959 pre-treatment into the VTA 5 min before fentanyl using both doses significantly and comparably reduced the opioid-induced GABA increase to the baseline levels. During the two last hours of microdialysis the accumbens GABA levels were oscillating around border significant increase in comparison to baseline concentrations (around 110% maximum). For the lower JMV2959 dose: JMV2959 2 µg/VTA + fentanyl 30 µg/kg vs. Ringer’s solution/VTA + fentanyl 30 µg/kg: effect of group F1,10 = 63.9, *p* < 0.001; effect of time F9,90 = 18.3, *p* < 0.01; time × group interaction F9,90 = 10.1, *p* < 0.001. For the higher dose: JMV2959 10 µg/VTA + fentanyl 30 µg/kg vs. Ringer’s solution/VTA + fentanyl 30 µg/kg: effect of group F1,10 = 101.5, *p* < 0.001; effect of time F9,90 = 20.0, *p* < 0.01; time × group interaction F9,90 = 16.1, *p* < 0.001. Both doses of JMV2959/VTA with fentanyl 30 µg/kg showed very low but significant GABA increase only at the end of the third hour after fentanyl administration: JMV2959 2 µg/VTA + fentanyl 30 µg/kg vs. Ringer’s solution/VTA + saline: effect of group n.s.; effect of time F9,90 = 3.4, *p* < 0.01; time × group interaction F9,90 = 3.0, *p* < 0.01; maximum effect 109% of baseline; JMV2959 10 µg/VTA + fentanyl 30 µg/kg vs. Ringer’s solution/VTA + saline: effect of group n.s.; effect of time F9,90 = 3.4, *p* < 0.01; time × group interaction F9,90 = 2.8, *p* < 0.01; maximum effect 109% of baseline.

A single dose of JMV2959 2 µg/VTA similarly to saline had no effect on accumbens GABA. A single 10 µg/VTA dose of JMV2959 induced slight but significant increase of accumbens GABA: JMV2959 10 µg/VTA + saline vs. Ringer’s solution/VTA + saline: effect of group n.s.; effect of time n.s.; time × group interaction F9,90 = 2.3, *p* < 0.05; with maximum effect of 104% of baseline.

#### 2.3.3. Pre-Treatment with JMV2959 Administration into the NAC

Figure 6b illustrates the observed influence of ghrelin antagonist administered into the NACSh on the accumbens fentanyl-induced GABA increase. Again, the 30 µg/kg fentanyl effects on accumbens GABA were practically the same with both, pre-treatment with saline i.p. as well as Ringer’s solution into the VTA and the group with systemic saline pre-treatment of fentanyl (see Section 2.2.1) was used for testing the JMV2959 effects when administered into the NAC (mean of three baselines + effects of fentanyl) (saline + fentanyl 30 µg/kg vs. saline + saline: effect of group F1,10 = 105.2, *p* < 0.001; effect of time F9,90 = 46.4, *p* < 0.001; time × group interaction F9,90 = 45.0, *p* < 0.001; maximum increase 191% of baseline level).

Pre-treatment with both JMV2959 doses into the NAC significantly reduced the fentanyl-induced accumbens GABA increase to the baseline levels. Since 100 min after fentanyl administration the accumbens GABA levels stayed slightly significant above the baseline concentrations (112–116% maximum). For lower JMV2959 dose: JMV2959 lower dose 8 mM/15 min/NAC + fentanyl 30 µg/kg vs. saline + fentanyl 30 µg/kg: effect of group F1,10 = 76.1, *p* < 0.001; effect of time F9,90 = 33.6, *p* < 0.001; time × group interaction F9,90 = 53.7, *p* < 0.001. For higher JMV2959 dose: JMV2959 higher dose 40 mM/15 min/NAC + fentanyl 30 µg/kg vs. saline + fentanyl 30 µg/kg: effect of group F1,10 = 74.2, *p* < 0.001; effect of time F9,90 = 22.7, *p* < 0.001; time × group interaction F9,90 = 35.1, *p* < 0.001. Both JMV2959 pre-treatments with fentanyl induced slight but significant GABA increase only during the last/third hour after fentanyl administration in comparison to saline + saline: for lower dose: JMV2959 lower dose 8 mM/15 min/NAC + fentanyl 30 µg/kg vs. saline + saline: effect of group n.s.; effect of time F9,90 = 8.9, *p* < 0.001; time × group interaction F9,90 = 8.0, *p* < 0.001; for higher dose: JMV2959 higher dose 40 mM/15 min/NAC + fentanyl 30 µg/kg vs. saline + saline: effect of group n.s.; effect of time F9,90 = 3.8, *p* < 0.001; time × group interaction F9,90 = 3.3, *p* < 0.05.

Single lower as well as higher JMV2959 dose administered into the NACSh did not significantly influence the accumbens GABA and also administration of saline had no effect on accumbens GABA.

### 2.4. Additional Behavioural Assay

**Figure 7:**
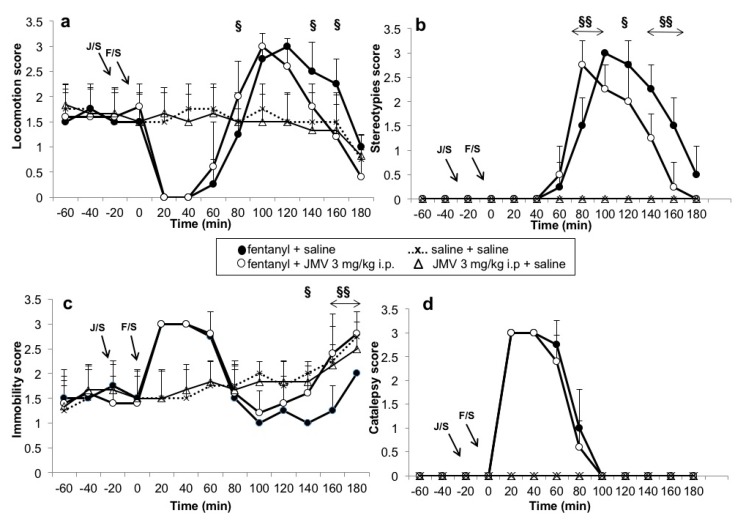
Effects of ghrelin receptor antagonist JMV2959 (3 mg/kg i.p.) on behavioural changes induced by fentanyl are illustrated in four monitored categories as means of behavioural scores (±SEM) separately: (**a**) locomotion; (**b**) stereotypies; (**c**) immobility; and (**d**) catalepsy. Behavioural changes during baseline period (intervals −60, −40, −20) and the pre-treatment with JMV2959/saline (0), are followed by 20–180 min of fentanyl/saline effects (20–180 min). The behavioural effects are illustrated as follows: saline + fentanyl (filled circle), JMV2959 + fentanyl (open circle), saline + saline practically identical with JMV 3 mg/kg + saline (cross with dotting). Differences between fentanyl and JMV2959 + fentanyl effects are expressed as ^§§^ *p* < 0.01, ^§^ *p* < 0.05. The horizontal arrows show intervals with appropriate significant changes (^§§^); the oblique arrows show the time of administration of J/S = JMV2959/saline or J/R = JMV2959/Ringer´s solution and F/S = fentanyl/saline.

As expected, fentanyl induced typical significant biphasic (inhibition-stimulation) behavioural changes in the rats, as illustrated in Figure 7, in accordance with our previous studies with morphine [16,17,21,85]. The fentanyl-induced changes were more dynamic in comparison with morphine and the behaviour returned to almost normal control parameters at the end of the experiment. We were interested in the ghrelin antagonist effects. JMV2959 pre-treatment 20 min before fentanyl induced moderate but significant changes within the fentanyl-evoked stimulation. In comparison to saline + fentanyl, in the JMV2959 pre-treated rats, we especially observed decreased locomotion, the fentanyl-induced behavioural stimulation was cut with JMV2959, the parameters of locomotion reached control levels 30 min earlier (between intervals 140 and 160 min) (see Figure 7a), although the fentanyl + saline locomotion was still significantly higher within 140 and 160 intervals in comparison to the control group (locomotion: saline + saline vs. fentanyl + saline time × group interaction F10,100 = 11.7; *p* < 0.05). The incidence of stereotypical behaviours (Figure 7b) was present within shorter period, it was lowered, but apparent (less confined gnawing and licking, almost no stereotyped sniffing). The rats started with gnawing and licking afterwards during still remaining stupor positions earlier than within the fentanyl + saline group, which shifted the beginning of occurrence of stereotypies. In comparison to fentanyl + saline, the pre-treatment with JMV2959 significantly increased the immobility scores (Figure 7c) during the last hour of the experiment (increased sedation) and during the last interval we observed sedation with eyes closed, similarly to the saline + saline group and correspondingly to the decrease in locomotion changes. In the fentanyl + saline rats, the immobility scores remained significantly lower within 140 and 160 intervals in comparison to control (immobility: saline + saline vs. fentanyl + saline time × group interaction F10,100 = 8.6; *p*< 0.05), but the JMV2959 pre-treated rats reached the scores of control group already before the interval 160 min. Catalepsy (Figure 7d) remained practically unchanged (JMV 3 mg/kg + fentanyl 30 µg/kg vs. saline + fentanyl 30 µg/kg: locomotion—effect of group n.s.; effect of time F10,100 = 44.0, *p* < 0.001; time × group interaction F10,100 = 3.03, *p* < 0.05.; stereotypies—effect of group F1,10 = 19.4, *p* < 0.05; effect of time F10,100 = 50.0, *p* < 0.001; time × group interaction F10,100 = 4.3, *p* < 0.001; immobility—effect of group F1,10 = 15.0, *p* < 0.01; effect of time F10,100 = 26.9, *p* < 0.001; time × group interaction F10,100 = 2.2, *p* < 0.05; catalepsy—effect of group n.s.; effect of time F10,100 = 47.0, *p* < 0.001; time × group interaction n.s.). During the last interval of the total 5 h microdialysis experiment, the control rats exhibited less locomotion parameters, increased immobility, sedation and closed eyes in comparison to the baseline intervals. The effects of single 3 mg/kg JMV2959 dose on the rat behaviour did not significantly differ from saline.

## 3. Discussion

Our results for the first time indicate significant involvement of GHS-R1As within the VTA and NAC in opioid/fentanyl-induced changes in the NACSh endocannabinoid AEA and 2-AG as well as GABA extracellular concentrations. Concerning endocannabinoids, the present results extent and particularize our previous study with morphine and systemic JMV2959 pre-treatment [17]. Fentanyl is considered as a µ-receptor-selective agonist, 100-fold more potent than morphine, mediating its reinforcing properties through both VTA as well as NAC µ-receptors [28,31,74]. The 30 µg/kg subcutaneous fentanyl used dose is about 300-fold lower than the morphine 10 mg/kg s.c. dose in our previous study and yet fentanyl induced much higher AEA increase in the NACSh (220%) in comparison to morphine (142% of baseline mean). However, the induced accumbens 2-AG decrease was practically comparable with fentanyl (81%) and morphine (85% of baseline). This indicates association of opioids-induced accumbens AEA efflux with their µ-receptor affinity. The observed opioid/fentanyl-induced accumbens AEA increase and 2-AG decrease are also in accordance with Vigano [54] and Caille [52].

Pre-treatment with JMV2959 in allF doses and types of administration (i.p., into the VTA or NAC) significantly changed the fentanyl-induced accumbens AEA increase and 2-AG decrease. The fentanyl-evoked AEA increase was reversed by pre-treatment with intraperitoneal 3 mg/kg dose of JMV2959, inducing a significant decrease. The fentanyl-induced 2-AG decrease was significantly deepened by the 3 mg/kg i.p. JMV2959. This is fully in accordance with our previous study with morphine and intraperitoneal JMV2959 pre-treatment [17]. In one experiment of our previous study [17], we have confirmed participation of central ghrelin system in the observed changes of AEA and 2-AG concentrations induced by opioid/morphine in the NACSh, when we co-administered ghrelin (40 µg/kg) with JMV2959 3 mg/kg i.p. before morphine, and ghrelin had abolished all the observed JMV2959 effects.

In the present study, we also observed a reverse of fentanyl-induced accumbens AEA increase when fentanyl s.c. was administered together with JMV2959 into the NACSh (perfusion with 8 or 40 mM for 15 min); however, the observed significant dose-dependent AEA decrease had sharper drop and shorter duration in comparison with the intraperitoneal JMV2959 effect, which was possibly due to the local (NAC) type of JMV2959 administration. The intra-VTA pre-treatment with JMV2959 (2 or 10 µg) prevented the fentanyl-induced accumbens AEA increase, but only the higher dose reversed the AEA levels to significant decrease. It is difficult to compare effects of differently administered various doses, but considering the effects of the pre-treatment JMV2959 doses, which per se/alone did not significantly influence the accumbens AEA, it can be suggested that GHS-R1As of both VTA and NAC brain structures participate in the significant JMV2959 reversal effects on the fentanyl/opioid-induced AEA increase in the NACSh, with major involvement of the NAC structure.

It has been described that predominant (but not selective) µ-receptor antagonist naltrexone did not attenuated ghrelin-induced food intake, locomotor stimulation and accumbens dopamine release [10,86], thus it has been suggested, that capability of reinforcement reduction through GHS-R1A antagonism does not include µ-receptors. It has also been described that subchronic JMV2959 (but not ghrelin) treatment significantly increased opioid peptide enkephalins/δ-agonists levels within the VTA and striatum and β-endorphin/κ-agonist levels within hippocampus and these changes are considered to contribute to the JMV2959/GHS-R1A antagonist-induced attenuation of opioid/morphine reinforcement [15]. To our knowledge, the influence of subchronic JMV2959 on µ-receptor endogenous ligands/β-endorphin has not yet been tested. However, our present results documented that GHS-R1A antagonism significantly affected the selective µ-opioid fentanyl-induced accumbens AEA changes which are believed to contribute to opioid reinforcement. Furthermore, in the present study surprisingly, all pre-treated JMV2959 doses and types of administration affected the fentanyl-induced AEA increase in a similar manner (mainly) without showing significant influence on the AEA levels “per se”. The JMV2959 3 mg/kg i.p. dose, doses administered into the VTA and lower dose into the NAC did not induce significant changes in accumbens AEA, although the higher 40 nM/NAC dose produced slight but significant AEA decrease (94% of baseline mean). Which corresponds with our previous study [17], when 6 mg/kg i.p. JMV2959 dose induced significant AEA drop to 92% of baseline and 3 mg/kg i.p. dose did not have significant influence. The above-summarized findings indicate presumable complexity of possibly several ghrelin involving neural pathways participating in the observed antagonism of fentanyl-induced accumbens AEA increase (and probably also in other here further presented changes). Possible cooperation of two or more neural systems and/or indirect effects might be considered. After all, such pattern is known from ghrelin influence of accumbens dopamine [87] and it also has been documented, that ghrelin orexigenic effect is dependent on several central networks, such as dopamine, cannabinoid, opioid and serotonine systems (see review [69]). In addition, the high constitutive activity of the GHS-R1A might possibly play some role [88]. Blocking of GHS-R1A by JMV2959 pre-treatments seem to prevent development of following fentanyl-induced ghrelin involving pathways and changes, which might at least partly contribute in massive pre-treatment effects using “per se” not effective doses. Further investigation is necessary to clarify the appropriate mechanisms. As mentioned earlier, it is thought that opioid-induced AEA increase in the NACSh possibly contribute to the opioid reinforcement through CB1 receptor-mediated process independent of dopamine [46,49,52]. Anandamide had been intravenously self-administered by squirrel monkeys, which proves its reinforcing properties [89]. Thus, the observed dose-dependent reversal of AEA increase evoked by opioids in the NACSh, which was caused by ghrelin antagonist, suggests an important contribution of central ghrelin system in the assumed anandamide impact to the opioid reinforcement. Our results indicate, that the GHS-R1A receptors within the NACSh as well as VTA both participate significantly in the opioid-induced accumbens anandamide increase, with possibly emphasized impact of the NACSh ghrelin signalling.

Similar to the 3 mg/kg JMV2959 i.p. administered effects, intra-accumbens administration of JMV2959 (perfusion with 8 or 40 mM for 15 min) also deepened significantly and dose-dependently the fentanyl-induced 2-AG accumbens decrease. When JMV2959 was administered into the VTA (2 or 10 µg), only the higher JMV2959 dose significantly deepened the fentanyl-induced 2-AG decrease. The lower JMV2959/VTA dose attenuated but prolonged the fentanyl-induced accumbens 2-AG decrease. Thus, both types of JMV2959 administration significantly changed the fentanyl-induced accumbens 2-AG decrease, but the pre-treatment effects seemed more expressed with the administration into the NACSh, similarly to the AEA. Analogously, the noticeable JMV2959 pre-treatment effects on fentanyl-evoked accumbens 2-AG decrease were induced using mainly per se not effective doses, with exception of the 40 mM/NAC JMV2959 dose, which induced significant 2-AG decrease to 97% of baseline mean, similarly to our previous study, when 6 mg/kg i.p. JMV2959 induced 2-AG decrease to 93% of baseline [17]. Again, possible complex ghrelin involving mechanisms/pathways should be considered participating in the predominant intensification of fentanyl-evoked accumbens 2-AG decrease induced by GHS-R1A antagonist pre-treatment. Further investigation is necessary. However, the significance of 2-AG decrease induced by acute opioid administration is so far difficult to interpret, as well as the observed intensified decrease with ghrelin antagonist. Recently considered and documented important role of 2-AG in reinforcement and addiction processes within the mesolimbic system has been mainly associated with chronic drug use (including opioids), changed motivational states, cue-evoked reward seeking, withdrawal and synaptic plasticity and also 2-AG increase [90,91].

GHS-R1A, CB1 and µ-opioid receptors are expressed within the NACSh as well as the VTA, thus interaction among the appropriate signalling systems within these brain structures could be considered above [5,37,38,42,50,92]. However, further investigation is necessary to elucidate the actual purpose and consequences of their possible relationships, including our findings mentioned The present data together with our previous results [16,17] associated with endocannabinoids and ghrelin signalling involvement in the opioid reinforcement, suggest that ghrelin signalling is possibly significantly involved in both dopamine-dependent as well as dopamine-independent opioid reinforcing processes in the NACSh. Midbrain GHS-R1As, co-localized with dopaminergic and cholinergic receptors [5,93], functionally interact in amplification of the dopaminergic signalling in the VTA neurons and stimulate accumbens dopamine efflux [86,94]. Indeed, the ability of ghrelin antagonism to decrease opioid-induced accumbens shell dopamine increase has been documented previously [15,16]. The brain endocannabinoid system is important for regulation of dopamine signalling during reinforcement processes [61,95]. Anandamide, when administered intravenously, also increased dopamine in the NACSh [61] as a neurochemical effect common to rewarding stimuli. The CB1 antagonist SR141716A infused into the NACSh significantly attenuated intravenous self-administration of heroin [49], but systemic SR141716A pre-treatment had no effect on morphine-induced dopamine increase in the NACSh [46,51]. Thus, accumbens CB1 receptors possibly significantly modulate opioid reinforcing properties through dopamine-independent mechanisms [52]. Thus, the significant reduction/reversal of opioid/fentanyl-induced accumbens AEA increase caused by JMV2959 might indicate an important participation of ghrelin signalling in the presumed non-dopaminergic opioid reinforcing mechanisms. Considering possible interactions between endocannabinoids and central ghrelin signalling, functional cooperation of CB1/endocannabinoids and GHS-R1A/ghrelin within hypothalamus has been described, which possibly contribute to ghrelin orexigenic effects, but similar link within NACSh to our knowledge has not been evidenced [66,67,68,69,72]. Further research is required to elucidate the appropriate involved mechanisms.

It has been suggested, that the opioid-induced GABA efflux in the NACSh [78,79] contributes to the opioid reinforcing properties [29,80]. The µ-opiod receptors are strategically located to modulate GABA release in the NAC, because they are expressed on medium spiny projection neurons (from VTA) and spiny interneurons in the NAC [27,96,97]. The majority of neurons within NAC are GABAergic medium spiny neurons (MSNs) [98] that send their output to several brain structures including the VTA [99] and ventral pallidum [100]. These GABA neurons receive inputs from the VTA (dopaminergic) and from hippocampus, amygdala and medial prefrontal cortex (glutamatergic) [101,102,103]. The VTA GABA neurons, which project to the NAC synapse largely onto cholinergic interneurons [104]. The simultaneous activation of µ and GABA-A receptors, which are co-expressed on GABAergic interneurons in the NAC markedly suppresses GABA release onto dopamine nerve endings, thus disinhibiting/enhancing dopamine efflux [29]. Alternatively, dopamine release may be evoked indirectly through activation of GABA-A and µ receptors on GABAergic medium spiny projection neurons in the VTA [27,28]. In either case, GABA elevated concentrations potentiate opioid-induced accumbens dopamine release. In the present study, we have demonstrated for the first time that pre-treatment with ghrelin antagonist in all given doses and types of administration prevented the opioid/fentanyl-induced accumbens GABA efflux in a very similar way. These findings suggest that ghrelin antagonism may inhibit opioid-induced dopamine release, which has been described previously [15,17], at least in part, by attenuating opioid-evoked GABA release in the NACSh. Since we have found comparable effects of JMV2959 administered into the VTA, the NAC and i.p. onto the fentanyl-induced accumbens GABA increase, possibly both modulatory mechanisms within the NAC and VTA participate on these ghrelin antagonist effects. These new findings further support the presumed complexity of possibly several ghrelin involving pathways which contribute to opioid reward and reinforcement.

It has been also established, that opioids’ activation of opioid receptors in the NAC decreases GABA release in their major projection area the ventral pallidum, generating dopamine-independent biological effects. It has been well documented, that CB1, µ as well as GHS-R1As are present on GABA presynaptic terminals in various brain structures [28,42,81,84]. CB1 receptors are located on inhibitory inputs to GABAergic medium spiny projection neurons in the NAC and it has been suggested that endocannabinoids release evoked by depolarization in the NAC and from the VTA dopaminergic neurons may act as retrograde messengers on reachable receptors of GABAergic afferents [65]. It was also described that VTA GABA-A/GABAergic system importantly contributes to the dopamine-independent opioid reinforcement [81,105]. Thus, the observed opioid-induced accumbens GABA increase might also contribute to dopamine-independent opioid reinforcement mechanisms, possibly involving endocannabinoids or ghrelin. Recently, important interactions between central ghrelin and GABAergic systems have been implicated within the CeA [83] and hypothalamus [84], hereby we have observed significant ghrelin and accumbens GABA interaction. Assuredly, further investigation of the particular involved mechanisms is necessary.

The additional behavioural assay confirmed that ghrelin antagonism moderately but significantly attenuated the fentanyl-induced behavioural stimulation (locomotion, stereotypical sniffing, partly also confined gnawing), which are considered as a sign of activation of nigrostriatal pathway [36,106]. It seemed that JMV2959 i.p. pre-treatment slightly speeded up onset of stimulation phase of fentanyl-induced biphasic behavioural changes, reduced especially fentanyl-induced increased incidence of walking, rearing and stereotypical sniffing and generally accelerated return of the fentanyl changed behaviour (locomotion and immobility) to the normal/control condition. In our previous study, JMV2959 in 1, 3 and 6 mg/kg i.p. single doses did not significantly influence rat locomotor activity 25 min after the JMV2959 administration, when monitored for 20 min using Ethovision program (Noldus, Wageningen, The Netherlands) [21].

Our results signify a strong participation of accumbens endocannabinoids, particularly anandamide, but also GABA in the neural opioid/fentanyl reinforcing processes and suggest that ghrelin antagonism may play an important role in the NACSh endocannabinoid/AEA and GABA changes possibly related to opioid/µ-receptor agonist reinforcement. Although GHS-R1A receptors within both NACSh as well as VTA have been found to contribute significantly to these effects, administration of ghrelin antagonist into the NACSh seemed to have stronger impact on the accumbens endocannabinoid opioid-induced changes. Collectively, our results indicate significant engagement of central ghrelin in GABA and endocannabinoid mechanisms in opioid/fentanyl reinforcement and encourage further investigation to assess, if ghrelin antagonism or substances affecting GABA or endocannabinoid concentrations and actions, such as GHS-R1A antagonists, can be used to prevent opioid/fentanyl-seeking behaviour.

## 4. Materials and Methods

### 4.1. Animals

Male Wistar rats (adult 8 weeks old; 200–250 g; Velaz, Praha-Lysolaje, Czech Republic) in groups of 6 were used. The animals were housed in polycarbonate cages and given free access to food and water with room temperature (22–24 °C), constant humidity (50–60%), and a 12-h light/dark reversed cycle for at least 7 days before the experiments, which were performed from 8 am to 3 pm during the dark period. Procedures involving animals and animal care were conducted in compliance with international laws; protocols respected EU Directive (2010/63/EU, 22 September 2010) and the Guidelines of the European Union Council (86/609/EU, 24 November 1986) and followed the instructions of the National Committee for the Care and Use of Laboratory Animals. Experiments were performed in accordance with the Animal Protection Act of the Czech Republic (No. 246/1992 Sb, 15 April 1992) and were authorized by the Expert Committee for Protection of Experimental Animals of the Third Faculty of Medicine, Charles University in Prague.

### 4.2. Drugs and Chemicals

**Figure 8:**
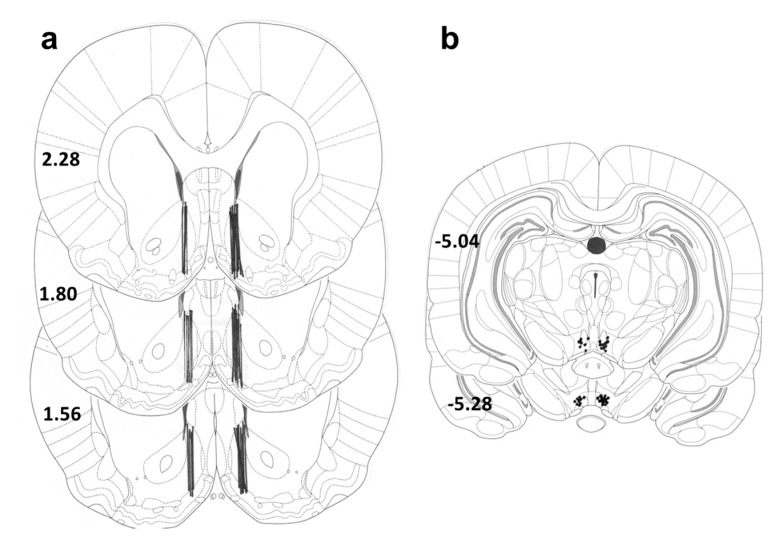
Schematic locations of: dialysis probes in the nucleus accumbens shell (**a**); and sites of infusions into the ventral tegmental area (**b**). Schematic locations of probe tips in animals which were involved in analyses of accumbens neurotransmitter concentrations (the bold lines indicate the dialyzing positions on the Figure 7a) and locations of JMV2959/Ringer’s solution administrations into the VTA (dark dots in the lower part of slices on the Figure 8b) as described in the atlas of Paxinos and Watson [113]. The distance from bregma (in mm) is indicated on the left of each schematic.

Fentanyl citrate was purchased from Sigma-Aldrich (St. Louis, MO, USA). JMV2959 (1,2,4-triazole derivate), which has been proved to be an GHS-R1A antagonist [18], was provided by Anton Bespalov (AbbVie, Heidelberg, Germany). All reagents were analytical grade. Fentanyl (30 µg/kg) was always dissolved in saline and administered subcutaneously (s.c.) 0.1 mL/100 g of body weight and saline was used as placebo; the chosen dose was selected following the literature as reliable analgesic and discriminative dose increasing accumbens dopamine [19,107,108]. JMV2959 was dissolved in saline, when administered intraperitoneally (i.p.) 20 min before fentanyl; the selected dose 3 mg/kg JMV2959 s.c. was determined based on our previous studies in Wistar rats [16,17] and the literature [109,110]. The dose 3 mg/kg JMV2959 had no effect on the rat behaviour. The intracerebral JMV2959 doses were in accordance with the literature [111,112]. When JMV2959 was administered intra-cerebrally, JMV2959 was dissolved in the Ringer’s solution (adjusted to pH = 7.0) and Ringer’s solution was used as a placebo. Doses 2 or 10 µg of JMV2959 were administered into the VTA at a volume of 0.5 µL for 1 min; the cannula stayed in place for another minute and after was retracted (5 µL microsyringe; Innovative Labor System, Stutzerbach, Germany). The administration sites were verified following the end of the experiment (Figure 8b), and only animals with correct injection sites were included in the statistical analysis. We have used the dialysis probe for administration of JMV2959 into the NAC. During perfusion with Ringer’s solution (always 2 µL/min) the inlet tube was switched to tube filled with 8 mM or 40 mM solution of JMV2959 in the Ringer’s solution for 15 min, starting 5 min before fentanyl administration; thereafter, the inlet tube was switched back to Ringer’s solution. The position of each dialysis probe was histologically verified (Figure 8a) after the completion of each microdialysis experiment and only animals with correct probe positions were included into the statistical evaluations.

### 4.3. In Vivo Microdialysis

The acute effects of fentanyl in rats were monitored after pre-treatment with JMV2959 or saline/Ringer’s solution, using the in vivo microdialysis model in the nucleus accumbens shell (NACSh). In separate groups, JMV2959 (3 mg/kg) was administered intraperitoneally or into the VTA (2 or 10 µg) or into the NAC (8 or 40 mM/15 min perfusion). After three baseline samples collecting, JMV2959 was administered intraperitoneally 20 min before fentanyl (s.c.). After four baseline samples collecting, JMV2959 was administered into the VTA 5 min before fentanyl or the NAC was perfused with JMV2959 for 15 min, starting 5 min before fentanyl. The dialysis samples were collected for a total of 260 min at 20 min intervals. Dialysate levels of AEA, 2–AG and GABA were analysed using high-sensitivity liquid chromatography combined with mass spectrometry.

#### 4.3.1. Surgery

The method is described in detail in Sustkova-Fiserova et al. [16,17]. Under ketamine—xylazine surgical anaesthesia (ketamine 100 mg/kg i.p., Narketan, Vetoquinol; xylazine 10 mg/kg i.p., Xylapan, Vetoquinol), using a stereotaxic instrument (Stoelting Co., Wood Dale, IL, USA), a disposable dialysis guide cannula (MAB4 probes, Agnthos, Lidingo, Sweden) was implanted in rats into the nucleus accumbens shell (NACSh coordinates—anterior (A): +2.0 mm and lateral (L): ±1.2 mm from bregma and vertical (V): 6.2 mm from occipital bone) [113]. The cannula was then secured to the skull with dental cement and an anchoring screw. The guide was randomly assigned to the left or right side. In experiments, were JMV2959/Ringer’s solution was administered into the VTA, two guide cannulas were implanted together on the same site, one into the NACSh (coordinates see above) and one into the VTA (VTA: A: −5.3 mm and L: ±0.8 mm from bregma and V: 8.2 mm from the skull) (unilaterally). The used coordinates to target the VTA district linked with ghrelin-involving food/drug motivation are chosen following literature [7,12]. Postoperative, the rats were housed in their own individual cages. After the end of the microdialysis experiments, the placements of the dialysis probe (NACSh) as well as placement of the infusion cannula (VTA) were verified histologically (Figure 8). Only animals with correct probe/cannula placement were used for subsequent statistical analysis.

#### 4.3.2. Microdialysis and Chemical Analysis Assay

In accordance with Sustkova-Fiserova [16,17], 48 h after implantation, a probe (MAB4, 2 mm active cuprophane membrane, Agnthos, Sweden) was inserted into the guide cannula and the probe was flushed with artificial cerebrospinal fluid (Ringer’s solution; 147 mM NaCl, 2.2 mM CaCl_2_ and 4.0 mM KCl; pH 7.0) at a constant rate of 2.0 μL/min (Univentor 864 Syringe Pump, Agnthos); we used dual swivels (Agnthos). After 60 min of habituation (when the dialysate was discarded), 20 μL samples were collected in small polyethylene tubes at 20-min intervals; the other 20-μL part of each interval dialysate served for other neurotransmitters detection. After three consecutive baseline samples, rats were injected with saline or JMV2959 (i.p.), which was followed (20 min later) by fentanyl or saline (s.c.) injection (in separate experiments). In experiments with JMV2959 administration into the VTA/NAC we have collected four baseline samples and 5 min before fentanyl, JMV2959 was administered into the VTA or NAC perfusion started and samples were further collected starting with fentanyl administration. Sampling continued for 3 h following injection of fentanyl or saline. Immediately after sampling, the samples were frozen at −70 °C. The amount of AEA, 2-AG and GABA in the dialysate were quantified using high-performance liquid chromatography combined with mass spectrometry (HPLC-MS). The appropriate HPLC-MS determination methods were described in detail earlier [17,114]. Thus, here only brief explanation: determination of GABA and endocannabinoids in the dialysate consisted of lyophilization in freeze dryer (Labconco Free Zone, Kansas City, MO, USA) to concentrate the substances from the dialysates, and detection using liquid chromatography combined with electrospray ionization tandem mass spectrometry (LC–ESI-MS/MS) which consisted of a chromatograph Accela 1250 (Thermo Scientific, Waltham, MA, USA), autosampler Accela (Thermo Scientific) and a TSQ Vantage mass spectrometer (Thermo Scientific). The data were acquired and processed using Xcalibur 2.1.0 software (Thermo Scientific). The in vitro recovery (probe MAB4, 2 mm, Agnthos) of anandamide (AEA) and 2-AG has been determined in our previous study [17]; the average recovery of AEA was 51 ± 4% and for 2-AG 53 ± 5%. However, detected extracellular concentrations from the NACSh dialysates oscillated around 0.9–3.0 ng/mL of anandamide and 0.1–0.7 ng/mL of 2-AG. The limit of quantification (LOQ) for AEA was 240 pg/mL and the LOQ for 2-AG was 280 pg/mL. In our previous study [115] has been described, that GABA efflux in the NACSh in our microdialysis experiments is Ca^2+^ dependent which indicates, that GABA extracellular concentration in the dialysates reflects the overflow of neuronally/from the synapses released neurotransmitter.

### 4.4. Additional Behavioural Assay

During the experiment, when 3 mg/kg JMV2959 was administered intraperitoneally 20 min before fentanyl, similarly to our previous studies with morphine [16,17,21,85], rats’ behavioural changes were monitored simultaneously in the course of microdialysis measurements. The following behavioural categories were monitored: immobility (eyes closed, akinesia, reduced responsiveness to environmental cues, sedation), catalepsy (exophthalmos, trunk rigidity frozen postures), locomotion (non-stereotyped activity, walking, rearing, grooming, sniffing), stereotypical behaviours (licking, stereotypical sniffing, confined gnawing) as was described previously in Sustkova-Fiserova et al. [17] (and in accordance with [116,117]. An observer, who was blinded to the treatment, scored all the behavioural categories at each microdialysis interval (every 20-min). The intensity or incidence of any behavioural changes which occurred during each/the whole 20 min interval were assessed using predefined anchor points on a 4 point scale: 0 = absent/no incidents; 1 = mild/1–5 incidents; 2 = moderate/medium/6–10 incidents; 3 = marked/maximum/more than 11 incidents. Behavioural changes were scored during the entire microdialysis period: 60 min baseline + 20 min pre-treatment and 3 h following fentanyl or saline administration.

### 4.5. Statistical Analysis

Raw data for endocannabinoids and GABA, not corrected for probe recovery, expressed as ng/mL/sample, were transformed into a percentage of baseline levels (mean of three or four intervals prior to pre-treatment). In addition, changes in behavioural parameters, within the 20-min intervals, were analysed. We used Sigma Plot 13 (Systat Software, Inc., San Jose, CA, USA) for statistical evaluation of the time course neurochemical and behavioural data. For statistical differences between the treatment groups (JMV2959 + fentanyl), (saline/Ringer’s solution + fentanyl), and (saline/Ringer’s solution + saline) relative to time-related changes during the in vivo microdialysis experiment, we have used two-way analysis of variance for repeated measures (ANOVA RM analysis) followed by Bonferroni multiple comparisons procedure. The group of animals was entered as the between-group factor and the time-points as repeated within-subject measures, we compared all treatments to baseline mean; 20-min intervals over 200–180 min of post-treatment. All statistical tests were performed as two-sided at a significance level of 0.05 (the *p* values of <0.05, <0.01 and <0.001 defined statistical significance). Groups of 6 animals were used; results are presented as the mean ± SEM.

## Abbreviations

2-AG	2-arachydonoylglycerol
AEA	*N*-arachidonoylethanolamine
CeA	central nucleus of amygdala
GABA	γ-aminobutyric acid
GHS-R1A	growth hormone secretagogue receptor A1
MSNs	medium spiny neurons
NAC	nucleus accumbens
NACSh	nucleus accumbens shell
VTA	ventral tegmental area
